# Risk perception of non-communicable diseases: A systematic review on its assessment and associated factors

**DOI:** 10.1371/journal.pone.0286518

**Published:** 2023-06-01

**Authors:** Miaw Yn Jane Ling, Norfazilah Ahmad, Azimatun Noor Aizuddin

**Affiliations:** Department of Public Health Medicine, Faculty of Medicine, Universiti Kebangsaan Malaysia, Cheras, Kuala Lumpur, Malaysia; Kurdistan University of Medical Sciences, ISLAMIC REPUBLIC OF IRAN

## Abstract

**Background:**

The burden of non-communicable diseases (NCDs) is increasing. Risk perception of NCDs is an important factor towards the uptake of preventive health interventions. There are various questionnaires assessing risk perception of NCDs, but no internationally standardized questionnaire has been available. Identification of factors associated with risk perception of NCDs may facilitate the development of targeted interventions. This systematic review aims to identify available questionnaire assessing risk perception of NCDs and the factors associated with risk perception of NCDs.

**Methods:**

The reporting of this systematic review is in accordance with the Preferred Reporting Items for Systematic Reviews and Meta-Analyses guidelines. We carried out a literature search through three databases (Scopus, PubMed, Web of Science) and targeted original article published in English between 2012 and 2021. Quality appraisal of the eligible articles was conducted using the Mixed Methods Appraisal Tool. Findings were synthesized using content analysis.

**Results:**

A total of 86 studies were included. We found a variety of questionnaires assessing risk perception of NCDs, with many differences in their development, domains, items and validity. We also identified several personal, sociopsychological and structural factors associated with risk perception of NCDs.

**Limitations:**

Most of the included studies were of cross-sectional design, and therefore the quality of evidence was considered low and exhibit a high risk of bias. The role of publication bias within this systematic review should be acknowledged as we did not include grey literature. Additionally, language bias must be considered as we only included English-language publications.

**Conclusion:**

Further development and testing of available questionnaire is warranted to ensure their robustness and validity in measuring risk perception of NCDs. All the identified factors deserve further exploration in longitudinal and experimental studies.

## Introduction

According to the World Health Organization (WHO), non-communicable diseases (NCDs) are chronic diseases which tend to have long duration. The main types of NCD include cardiovascular diseases (CVDs), cancers, chronic respiratory diseases and diabetes (DM) [1]. NCDs kill 41 million every year, which is equivalent to 71% of worldwide deaths. The total annual number of NCD deaths is expected to increase to 55 million if the current trend continues [2]. Study also reported that NCDs impose a great impact on both households and impoverishment globally, at all levels of development [3]. Furthermore, the rising national health-care budget and the loss of productivity or ability to work due to NCDs pose serious and growing threats to many nations’ economic stability [4].

In general, NCDs occur due to the combination of several factors, including genetic, physiological, environment and behavioural factors [1]. Behavioural factors such as harmful use of alcohol, physical inactivity, salt intake and tobacco use are modifiable [5]. An individual’s behaviour is determined by many factors. According to the Theory of Planned Behaviour, behaviours are determined by behavioural intentions [6], while Social Leaning Theory posits that behaviour is determined by expectancies, incentives and self-efficacy [7]. Additionally, the Health Belief Model (HBM) hypothesizes that health-related behaviour depends on risk perception [7]. The term “risk perception” is defined as an individual’s subjective judgement of the risk related to some event [8].

According to the HBM, there are four types of risk perception, including perceived susceptibility, perceived severity, perceived benefits and perceived barriers. Perceived susceptibility refers to the subjective risk of contracting a health condition, while perceived severity is the judgement on degree of seriousness of a given health condition. In addition, perceived benefits refers to an individual’s estimate that a given behaviour will be effective in reducing the threat of disease, while perceived barrier refers to the barriers that must be overcome to follow the health recommendation [7, 9]. An individual’s perceived risk of developing a disease is an important factor towards adopting healthy lifestyles and the uptake of preventive health interventions [10].

Many studies have been conducted to assess the perceived risk of developing NCDs. The use of questionnaire as an instrument to measure an individual’s risk perception of NCDs has been widely adopted [10, 11]. The reason for such wide adoption is that the use of questionnaire is a feasible and inexpensive method for data collection. This method can also be used on a large number of respondents, enabling statistical analysis that give more significant power than other methods [11]. A robust questionnaire is essential in ensuring reliable and valid measurement of risk perception of NCDs. Hence, validity of questionnaire is an important consideration when choosing an instrument for assessing risk perception of NCDs.

To better understand risk perception of NCDs, identification of its associated factors is essential. Literatures have highlighted several factors associated with risk perception of certain disease, including personal factor variables [demographic: age, ethnicity, income; lifestyle: smoking; health history: body mass index (BMI), family history of the disease] [9, 12–14] sociopsychological variables (cognitive factor, affective factor, personality and coping, perception of own health) [9, 13, 14] and structural variables (knowledge about the disease, prior contact with the disease) [9, 15].

Despite the potential usefulness of the available questionnaires for assessing risk perception of NCDs, to our knowledge, there are no systematic reviews that summarize these instruments and assess their validity. Previous systematic review also focused on factors that specifically influence risk perception of certain disease, such as cancer [14], while literatures synthesizing data regarding factors influencing several major NCDs are lacking. Therefore, the objective of this systematic review was to identify available questionnaire for assessing risk perception of NCDs and to characterize the existing literatures on factors associated with risk perception NCDs. This synthesis will assist researchers in selecting the most appropriate instrument for assessing risk perception of NCDs, and form an empirically-grounded conceptual framework for future NCD risk communication research.

## Materials and methods

The reporting of this systematic review is in accordance with the Preferred Reporting Items for Systematic Reviews and Meta-Analyses (PRISMA) 2020 statement [16] (S1 Appendix). The outcomes which are not related to patient or clinical outcome prevented the protocol to be included in the PROSPERO registration [17].

### Research question formulation

The review question was developed using the PEO (population, exposure, outcome) concept [18]. The PICO (population, intervention, comparator, outcome) framework is commonly used in assessing the effectiveness of an intervention or therapy. As this review will focus on the relationship between certain risk factors and a health outcome, the use of PEO concept is recommended [18]. In this systematic review, population refers to general population, exposure refers to associated factors of risk perception of non-communicable disease and outcome is risk perception of non-communicable diseases. The main review questions are: (1) Is there any available questionnaire to assess risk perception of non-communicable diseases? (2) What are the factors associated with the risk perception of non-communicable diseases?

### Data source and search strategy

**Fig 1:**
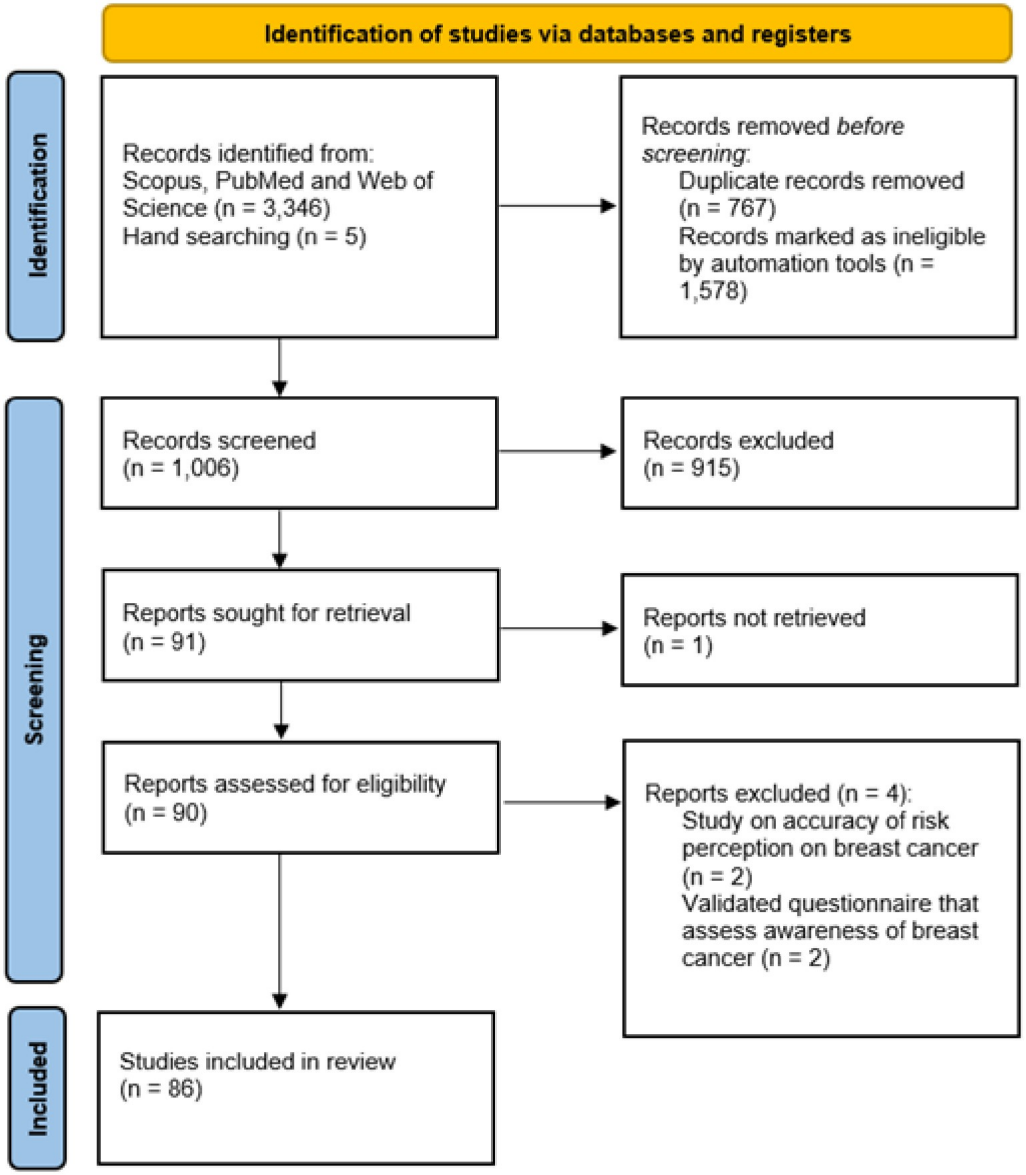
PRISMA flow diagram.

The literature search was conducted in December 2021, including three databases: Scopus, PubMed and Web of Science. In addition, hand searching of the reference lists of included studies was undertaken to identify further relevant references. The keywords used for the searching of related articles are provided in Table 1. There were 3,346 records identified from the three databases and 5 records identified from hand searching. Automated tools were used and 1,578 record were excluded based on year, publication type and language. A total of 767 duplicate records were found and removed, leaving 1,006 records for title screening (Fig 1). The records were exported from the databases into an Excel sheet for screening.

**Table 1:** Keywords used in the screening process.

Database	Search string
Scopus	1) TITLE-ABS-KEY(("questionnaire" OR "instrument" OR "tool") AND ("risk perception" OR "perceived risk") AND ("non-communicable disease*" OR "noncommunicable disease*" OR "NCD" OR "chronic respiratory disease*" OR "chronic obstructive pulmonary disease*" OR "asthma" OR "hypertension" OR "diabetes" OR "diabetes mellitus" OR "cancer" OR "neoplasm", "cardiovascular disease" OR "heart attack" OR "stroke")) 2) TITLE-ABS-KEY((“associated factor*” OR “risk factor*” OR “association*” OR “determinant*” OR “predictor*”) AND (“risk perception” OR “perceived risk”) AND ("non-communicable disease*" OR "noncommunicable disease*" OR "NCD" OR "chronic respiratory disease*" OR "chronic obstructive pulmonary disease*" OR "asthma" OR "hypertension" OR "diabetes" OR "diabetes mellitus" OR "cancer" OR "neoplasm", "cardiovascular disease" OR "heart attack" OR "stroke"))
Web of Science	1) TS = (("questionnaire" OR "instrument" OR "tool") AND ("risk perception" OR "perceived risk") AND ("non-communicable disease*" OR "noncommunicable disease*" OR "NCD" OR "chronic respiratory disease*" OR "chronic obstructive pulmonary disease*" OR "asthma" OR "hypertension" OR "diabetes" OR "diabetes mellitus" OR "cancer" OR "neoplasm", "cardiovascular disease" OR "heart attack" OR "stroke")) 2) TS = ((“associated factor*” OR “risk factor*” OR “association*” OR “determinant*” OR “predictor*”) AND (“risk perception” OR “perceived risk”) AND ("non-communicable disease*" OR "noncommunicable disease*" OR "NCD" OR "chronic respiratory disease*" OR "chronic obstructive pulmonary disease*" OR "asthma" OR "hypertension" OR "diabetes" OR "diabetes mellitus" OR "cancer" OR "neoplasm", "cardiovascular disease" OR "heart attack" OR "stroke"))
PubMed	1) ((questionnaire[Title/Abstract] OR instrument[Title/Abstract] OR tool[Title/Abstract]) AND (risk perception[Title/Abstract] OR perceived risk[Title/Abstract])) AND (non-communicable disease*[Title/Abstract] OR noncommunicable disease*[Title/Abstract] OR NCD[Title/Abstract] OR chronic respiratory disease*[Title/Abstract] OR chronic obstructive pulmonary disease*[Title/Abstract] OR asthma[Title/Abstract] OR hypertension[Title/Abstract] OR diabetes[Title/Abstract] OR diabetes mellitus[Title/Abstract] OR cancer[Title/Abstract] OR neoplasm[Title/Abstract] OR cardiovascular disease[Title/Abstract] OR heart attack[Title/Abstract] OR stroke[Title/Abstract]) 2) ((associated factor*[Title/Abstract] OR risk factor*[Title/Abstract] OR association*[Title/Abstract] OR determinant*[Title/Abstract] OR predictor*[Title/Abstract]) AND (risk perception[Title/Abstract] OR perceived risk[Title/Abstract])) AND (non-communicable disease*[Title/Abstract] OR noncommunicable disease*[Title/Abstract] OR NCD[Title/Abstract] OR chronic respiratory disease*[Title/Abstract] OR chronic obstructive pulmonary disease*[Title/Abstract] OR asthma[Title/Abstract] OR hypertension[Title/Abstract] OR diabetes[Title/Abstract] OR diabetes mellitus[Title/Abstract] OR cancer[Title/Abstract] OR neoplasm[Title/Abstract] OR cardiovascular disease[Title/Abstract] OR heart attack[Title/Abstract] OR stroke[Title/Abstract])

### Inclusion and exclusion criteria

The inclusion criteria were: (1) publication from 2012–2021; (2) publication in English language; (3) original article (4) described questionnaire that explicitly assessed risk perception of non-communicable diseases or questionnaire that contained at least five items measuring risk perception of non-communicable diseases, and 5) described associated factors of risk perception of non-communicable diseases. We limited the publication date to 2012 and 2021 (articles published in the past 10 years) so that we can build our systematic review on the recent literature considering the dynamic pattern of risk perception [19, 20]. Non-original articles such as conference proceedings, commentary, reports, review articles and systematic reviews were excluded.

### Study selection

Two authors (JLMY and NA) screened the titles and abstracts independently based on the review questions. A total of 915 articles were removed during the screening. For the remaining 91 articles, 90 full-text were retrieved for assessment of eligibility, while the remaining one article was inaccessible due to post-publication correction [21]. Disagreements were resolved through discussion with a third researcher (ANA) to reach consensus. 4 articles were removed as two articles focused on accuracy of risk perception on breast cancer [22, 23] and another two articles described the validation of questionnaire assessing awareness but not perception of breast cancer [24, 25], leaving a total of 86 articles to proceed with quality appraisal.

Quality appraisal was conducted by JLMY and ANA on all studies using the Mixed Methods Appraisal Tool (MMAT) [26]. The MMAT is a critical appraisal tool which is developed to appraise studies included in systematic mixed study reviews. The methodology quality of five categories of studies (qualitative study, randomized control trials, non-randomized studies, quantitative descriptive study and mixed methods study) can be appraised using this tool. For each category, five criteria are used to assess the quality of the study.

### Data extraction and synthesis

JLMY and NA extracted the data independently using a standardized data extraction form which is organized using Microsoft Excel. Information collected in the form include: (1) author, (2) publication year, (3) reference, (4) country, (5) study design, (6) statistical analysis and (7) results. Fig 1 shows the PRISMA flow diagram. Data synthesis was achieved using content analysis in order to condense text into fewer content-related categories [27].

## Results

### Background of the eligible studies

A total of 86 studies were included in this systematic review (Tables 2 and 3). Slightly less than half of the studies (n = 40) were conducted in United States of America (USA). Other studies were conducted in Belgium, Brazil, Canada, China, Denmark, Finland, Germany, Ghana, Hong Kong, Iran, Iraq, Italy, Jordan, Kenya, Korea, Malaysia, Mexico, Myanmar, Netherland, Nigeria, South Africa, Spain, Sweden, Switzerland, Taiwan, Turkey and the United Kingdom (UK). A total of 45 articles were published between the year 2012 to 2016, while 41 articles were published between 2017 to 2021.

**Table 2:** Characteristics of studies related to questionnaire assessing risk perception of NCDs (n = 14).

No	Author, Year	Study Country	Risk Perception Measurement	Study Population	Item Development	Language (developed & translated	No. of Items	Measure/Scale	Validation Analysis	Application Mode	Quality (%)
1	Adedeji et al. 2021 [28]	Nigeria	Prostate cancer	Male adults living in rural communities in Southwest Nigeria	Based on Health Belief Model	Developed: English Translated: Yoruba	26 (perceived threat:7; screening behaviour: 9; knowledge: 10)	5-point Likert	Face validity	Interviewer-administered	100
2	Hassen et al. 2021 [29]	Belgium	CVDs	Adults aged 18 years and older	Adapted from previous validated questionnaire	Developed: English Translated: Dutch	20 (risk perception: 7; perceived benefit & intention to change of physical activity: 6; perceived benefit & intention to change of dietary habit: 7)	4-point Likert	Exploratory factor analysis, confirmatory factor analysis	Self-administered (postal & online platforms)	80
3	Martínez-Urquijo et al. 2021 [30]	Spain	Breast cancer	Women without breast cancer	Adapted from previous studies	Developed: English Translated: Spanish	31 (risk factor knowledge: 9; knowledges of signs & symptoms: 9; perceived risk: 6; barriers: 7)	5-point Likert for perceived risk and barrier items	Exploratory factor analysis	Self-administered	40
4	Mya et al. 2021 [31]	Myanmar	CVDs, DM, cancer, chronic respiratory diseases	Participants from outpatient department of Yangon General Hospital (25–60 years and without NCDs)	Based on Health Belief Model	Developed: English Translated: Burmese	21 (perceived susceptibility: 4; perceived benefit: 5; perceived barrier: 3; self-efficacy: 5; behavioural change intention: 4)	4-point Likert	Exploratory factor analysis, confirmatory factor analysis	Not mentioned	100
5	Taylan et al. 2021 [32]	Turkey	Breast cancer	Women aged 20 and over without breast cancer	Based on literature review	Not mentioned	24 (perceived knowledge: 4; perceived treatment belief: 5; perceived need for health check: 4; perceived stigma: 4; perceived fear: 4; perceived risk: 3)	5-point Likert	Exploratory factor analysis, confirmatory factor analysis	Face-to-face interview	60
6	Anuar et al. 2020 [33]	Malaysia	Chronic kidney disease (CKD)	Patients with DM from outpatient clinics	Based on Health Belief Model	Developed: English Translated: Malay	61 (perception based on socio-psychology: 26; perceived benefit: 6; perceived barrier: 9; perceived susceptibility: 9; perceived severity: 5; cue to action: 6)	10-point Likert	Exploratory factor analysis, confirmatory factor analysis	Self-administered	80
7	Agarwal et al. 2019 [34]	Canada	High blood pressure, DM	Individual aged 55 and older	Based on previous surveys in Canada	Developed: English	48 (knowledge of CVD & DM: 18; current health behaviour: 13; health-related quality of life: 5; perceived risk & understanding:7; self-efficacy: 5)	Various measures (e.g. few response options, Likert scales, etc.)	Content validity, face validity, internal consistency, test-retest reliability	Self-administered	60
8	Alaa & Shah 2018 [35]	Iraq	Stroke, DM, hypertension, CVD, cancer	Adults aged 18–40 years old	Adapted from previous studies	Developed: English Translated: Arabic	30 (socio-demographic: 6; family history of chronic disease: 5; special practice:4; perceived severity: 5; information seeking: 5; disease probability: 5)	5-point Likert (except socio-demographic & family history of chronic disease)	Exploratory factor analysis, confirmatory factor analysis	Self-administered	60
9	Kaba et al. 2017 [36]	South Africa	Non-communicable diseases	Adults aged 20 years and above	Based on Health Belief Model	Not mentioned	35 (perceived susceptibility: 5; perceived severity: 5; perceived benefit: 7; perceived barriers: 7; perceived cues to action: 6; perceived self-efficacy: 5)	5-point Likert	Content validity	Not mentioned	60
10	Woringer et al. 2017 [37]	UK	Heart attack, stroke	Individual aged 40–74 years free of vascular disease	Based on Health Belief Model and Transtheoretical Model	English (no translation to other language)	26 (knowledge: 8; perceived risk: 8; perceived benefit: 7; healthy eating intention: 3)	4-point Likert & 5-point Likert	Exploratory factor analysis	Self-administered	80
11	Lin et al. 2016 [38]	Taiwan	Cervical cancer	Female adolescent from three colleges in Southern Taiwan	Based on literature review	Not mentioned	6-item human papillomavirus-related perceived risk scale	6-point Likert	Content validity, face validity	Self-administered	60
12	Morales-Sánchez et al. 2014 [39]	Mexico	Skin cancer	Mexican who attended an outpatient clinic and aged 18 years and above	Based on literature review and expert opinion	Spanish (translation not mentioned)	18 (affective aspects: 5; behavioural aspects: 5; severity: 3; susceptibility: 3; probability: 2)	7-point visual Likert	*t* test, chi-square test, Pearson correlation, exploratory factor analysis	Self-administered	60
13	Hafizah et al. 2013 [40]	Malaysia	Hypertension, DM, heart disease, cancer, stroke	Staff and clients of a health clinic (aged 18 years and above)	Based on expert opinion	Not mentioned	24 (special practice: 4; information seeking: 5; perceived probability of disease: 5; health-related behaviour: 5; perceived severity: 5)	5-point Likert except for perceived probability of disease (10- point Likert)	Exploratory factor analysis	Self-administered	60
14	Chan & Leung 2012 [41]	Hong Kong	Coronary heart disease	Adults aged 18 years and above	Based on literature review and expert opinion	English and Chinese (translation not mentioned)	9 (perceived seriousness: 4; perceived risk: 5)	5-point Likert	Content validity, face validity	Investigator-administered	40

**Table 3:** Characteristics of studies related to factors associated with risk perception of NCDs (n = 75).

No	Author, Year	Study Country	Statistical Analysis	Associated Factors of Risk Perception of NCDs			Outcome (type of NCD)	Quality (%)
				Personal Factors	Sociopsychological Factors	Structural Factors		
1	Adedeji et al. 2021 [28]	Nigeria	Descriptive, multiple regression and logistic regression	Prostate cancer (age, religion, income)			Prostate cancer	100
2	Fein et al. 2021 [42]	USA	Pearson’s chi-square test, Fisher’s exact test, Student’s t-test, analysis of variance (ANOVA)	Anal cancer (past sexually transmitted disease diagnosis, history of anoreceptive intercourse, male partner, human immunodeficiency virus/ HIV status)			Anal cancer	60
3	Grauman et al. 2021 [43]	Sweden	Descriptive, logistic regression analysis	CVD (family history of CVD, age, smoking)	CVD (self-perceived general health)		CVD	80
4	Osei et al. 2021 [44]	Ghana	Descriptive, chi-square analysis, multivariate binary regression analysis	Breast cancer (academic year, school, family history of breast cancer, use of oral pills/injectable contraception, history of breast cancer screening)	Breast cancer (intention to perform breast self-examination)	Breast cancer (knowledge of someone with breast cancer)	Breast cancer	80
5	Turner et al. 2021 [45]	Canada	Descriptive, Spearman correlation, Kruskal-wallis test, cumulative logistic regression	Lung cancer (age, gender, increased pack-decades smoked, former smoker, dyspnoea/cough, FEV%, history of chronic obstructive pulmonary disease, family history of lung cancer)			Lung cancer	60
6	Vornanen et al. 2021 [46]	Finland	Descriptive, chi-square, one-way anova, structural equation modeling	DM (physical activity, BMI, 2-hour glucose); CVD (BMI)			DM, CVD	100
7	Zarghami et al. 2021 [47]	Iran	Descriptive, simple and multiple linear regression, Spearman correlation	Lung cancer (age, gender, “years smoked”)			Lung cancer	80
8	Abshire et al. 2020 [48]	USA	Chi-square, logistic regression analysis	Perceived risk for gaining excess weight, hypertension and DM (BMI)			High blood pressure, DM, high cholesterol, heart disease, stroke, excess weight gain	60
9	Antwi et al. 2020 [49]	USA	Descriptive, Mann-Whitney U test, Pearson’s correlation, chi-square test	T2DM (BMI, fat mass percent, waist circumference, high-density lipoprotein level)			Type 2 diabetes (T2DM)	60
10	Khan et al. 2020 [13]	USA	Descriptive, univariate one-way analysis of variance, chi-square test, logistic regression	DM (age, BMI, family history with DM)	DM (self-perceived health)	DM (knowledge)	DM	80
11	Khlaifat et al. 2020 [50]	Jordan	Descriptive, t-test, ANOVA, Tukey’s post-hoc analysis	DM (family history of DM, caring for a relative with DM)			DM	80
12	Palmero et al. 2020 [51]	Brazil	Descriptive, Student’s t-test, ANOVA, Mann-Whitney, Kruskal-Wallis tests, chi-square, Fisher’s exact test, correlation coefficient of Spearman, Cronbach coefficient	Cancer (education, number of cancer deaths among first-degree relatives)		Cancer (risk discussion within the family)	Cancer	60
13	Russell et al. 2020 [52]	South Africa	Descriptive, chi-square analysis, Fisher’s exact test	Cervical cancer (age, income)			Cervical cancer	60
14	Stol et al. 2020 [53]	Netherland	Descriptive, two-tailed t-tests, chi-square, multivariable linear regression model	CVD, DM, CKD (family history of DM, family history of CVD, BMI, waist circumference, physical activity)			CVD, DM, CKD	60
15	Sulaiman et al. 2020 [54]	Malaysia	Descriptive, Mann-Whitney, chi-square tests	T2DM (gender)			T2DM	80
16	Gibson et al. 2019 [55]	USA	Descriptive, ANOVA, Pearson chi-square, multinomial logistic regression	Cervical cancer (age, age at first intercourse, family history)	Cervical cancer [perceived risk of human papillomavirus (HPV) exposure]	Cervical cancer (recommendation for pap smear by healthcare professional)	Cervical cancer	80
17	Guo et al. 2019 [56]	China	Descriptive, Pearson correlation, stepwise multivariate linear regression	DM (age, family history of DM)			DM	60
18	Hall et al. 2019 [57]	USA	Bivariate analysis, multivariable logistic regression	Breast cancer, colorectal cancer (ethnicity)			Breast cancer, colorectal cancer	100
19	Heidemann et al. 2019 [58]	Germany	Descriptive, logistic regression analysis	DM (age, family history of DM)		DM (informed about increased DM risk by physician)	DM	60
20	Hsueh et al. 2019 [59]	USA	T-tests, chi-square tests, logistic regression	DM (ethnicity)			DM	100
21	Pelullo et al. 2019 [60]	Italy	Descriptive, t test, chi-square test, generalized estimation equation analysis	DM (age, BMI, close relatives with DM, had chronic disease)	DM (perception of own health status)	DM (need of information about DM)	DM	100
22	Perez et al. 2019 [61]	USA	Chi-square test, Fisher’s exact test, two-sample t test, Wilcoxon rank-sum test, linear regression	Lung cancer or smoking-related disease (age, ethnicity, education, smoking)	Lung cancer or smoking-related disease (perceived benefit of quitting smoking, cancer worry)		Lung cancer, smoking-related disease	60
23	Turbitt et al. 2019 [62]	USA	Descriptive, bivariate logistic regression, multivariable logistic regression, bivariate multinomial logistic regression analysis	Breast cancer, colorectal cancer (genetic testing)		Breast cancer, colorectal cancer (genetic counselling)	Breast cancer, colorectal cancer	60
24	Chalian et al. 2018 [12]	USA	Descriptive, Pearson’s correlations tests, independent sample t-tests, paired t-tests, multi-group structural equation modeling	Cancer (ethnicity, age, income, smoking)	Cancer (insured status)		Cancer	80
25	Pasi et al. 2018 [11]	Malaysia	Descriptive, chi-square test, binary logistic regression	Cancer (family history of cancer)			Cancer	60
26	Skøt et al. 2018 [63]	Denmark	Independent sample t-test, chi-square test, Pearson correlation coefficients, hierarchical linear multiple regression analysis, binary logistic regression analysis	T2DM (BMI, sweet consumption, prior T2DM screening, physical activity, parental education, family history of T2DM)	T2DM (conscientiousness, emotional stability)		T2DM	60
27	Wilson et al. 2018 [64]	USA	Descriptive, Pearson’s chi-square tests, independent sample t tests, one-way ANOVA, binary logistic regression, multinomial logistic regression	Cervical cancer (diagnosed with HPV, family member with cervical cancer, unsure of family history of cervical cancer, more than one sex partner)	Cervical cancer (perceived risk of cervical cancer same or greater than others, support of HPV vaccine mandates, insured status)		Cervical cancer	60
28	Butler et al. 2017 [65]	USA	two-sample t test, chi-square test, multiple linear regression	Lung cancer (education, family history of lung cancer, smoking, living with smoker)			Lung cancer	60
29	Chopra & Chopra 2017 [15]	USA	Chi-square test, independent sample t-tests, multivariable logistic regression	DM (age, BMI, ethnicity, physical activity)	DM (personal control)	DM (knowledge)	DM	60
30	Desgraz et al. 2017 [66]	Switzerland	Chi-square, univariable and multivariable logistic regression	CVD (gender, age, hyperlipidemia, DM)			CVD	80
31	Kaba et al. 2017 [36]	South Africa	Descriptive, Pearson’s chi-square	NCDs (age)			NCDs	60
32	Kowall et al. 2017 [67]	Germany	Poisson regression	DM (age, gender, education, obesity, parental DM)	DM (self-rated general health)		DM	60
33	Osazuwa-Peters et al. 2017 [68]	USA	Descriptive, chi-square, independent samples t tests, univariate and multivariable linear regression analysis	Oropharyngeal cancer (ethnicity, education)			HPV-associated oropharyngeal cancer	60
34	Silverman et al. 2017 [69]	USA	Descriptive, logistic regression analysis	Cancer (family history of cancer, BMI)			Cancer	80
35	Taber et al. 2017 [70]	USA	Descriptive, linear regression analysis	Cancer (gender, age, ethnicity); CVD (age)			Cancer, CVD	80
36	Vandyke & Shell 2017 [71]	USA	Linear regression	Breast cancer (abnormal mammograms, family history with breast cancer)	Breast cancer (insured status)		Breast cancer	60
37	Yüksel et al. 2017 [72]	Turkey	Descriptive, Pearson’s chi-square, Spearman’s correlation, logistic regression analysis	Breast cancer (age, post-menopausal phase, income, family history of breast cancer, having regular breast self-examination and clinical breast examination)			Breast cancer	80
38	Basilio et al. 2016 [73]	USA	Independent t-test, chi-square test, t-test, correlational analyses	DM (ethnicity, ethnic identity, number of family members with DM)	DM (perceived similarity with other people that get DM)		DM, breast cancer	60
39	Boo et al. 2016 [74]	Korea	Descriptive, chi-square, Fisher’s exact test	CVD (taking medication for blood pressure control or blood lipid, family history of CVD)			CVD	60
40	Chen et al. 2016 [75]	USA	Descriptive, chi-square test, multinomial logistic regression, multivariate linear regression	Lung cancer (smoking)			Lung cancer	80
41	Joiner et al. 2016 [76]	USA	Descriptive, logistic regression analysis	DM (history of gestational DM, education)	DM (optimistic bias, worry, perceived risk to health or other chronic conditions)		DM	60
42	Osazuwa-Peters & Tutlam 2016 [77]	USA	Pearson’s correlation, chi-square, one-way ANOVA, binary logistic regression analysis	Oral cavity and oropharyngeal cancer (number of sexual partners, age at sexual debut)			Oral cavity and oropharyngeal cancer	60
43	Shah et al. 2016 [78]	USA	Descriptive, ANOVA, chi-square, Fisher’s exact test, confirmatory factor analysis	DM (age, gender, education level)			DM	60
44	Vornanen et al. 2016 [79]	Finland	Descriptive, chi-square, Pearson’s correlation, multivariate regression analysis	NCDs (family history, gender, age, education, smoking, alcohol, BMI, physical activity)			NCDs (CVD, DM, cancer, severe depression)	100
45	Amuta et al. 2015 [80]	USA	Descriptive, multiple linear regression analysis	T2DM (gender, family history of T2DM, BMI)			T2DM	60
46	Clark & Lavielle 2015 [81]	Mexico	Chi-square test, Student t-test	Osteoporosis (age, gender, family history of osteoporosis)			Osteoporosis	80
47	Fukuoka et al. 2015 [82]	USA	Descriptive, multiple logistic regression	DM (female, family history of DM, ethnicity, high cholesterol, high BMI); Heart attack (family history of heart attack, high blood pressure, high BMI)			DM, heart attack	80
48	Guess et al. 2015 [83]	UK	Descriptive, Student’s t-test, Spearman’s test			DM (being informed by health professional to be high risk)	DM	60
49	Hamilton & Lobel 2015 [84]	USA	Structural equation modeling analysis	CVD (family history, smoking, BMI); Breast cancer (BMI); Lung cancer (family history, smoking)	CVD (social exposure to CVD, perception of stigma by others, locus of control, optimistic); Breast cancer (perception of stigma by others); Lung cancer (perception of stigma by others, optimistic)		CVD, breast cancer, lung cancer	40
50	Kye et al. 2015 [85]	Korea	Chi-square test, binary logistic regression	Cancer (gender, age, income, family history of cancer)			Cancer	60
51	Peipins et al. 2015 [86]	USA	Path model analysis	Ovarian cancer (number of relatives with cancer)	Ovarian cancer (belief in the genetic cause of cancer)	Ovarian cancer (cancer knowledge)	Ovarian cancer	60
52	Piccinino et al. 2015 [87]	USA	Descriptive, logistic regression analysis	DM (BMI, family history of DM)		DM (been told by healthcare professional to be at risk)	DM	80
53	Rice et al. 2015 [88]	USA	Descriptive, ordered logistic regression	Cancer (gender, age, alcohol consumption, colon cancer screening)			Cancer	80
54	Temu et al. 2015 [89]	Kenya	Descriptive, chi-square, multivariate logistic regression	CVD (gender)			CVD	60
55	Godino et al. 2014 [90]	UK	Descriptive, Spearman’s rank correlation coefficient, linear regression, logistic regression, multivariable regression	T2DM (body fat percentage)	T2DM (self-perceived health, DM-related worry)		T2DM	60
56	Lucas-Wright et al. 2014 [91]	USA	Chi-square test, multiple multinomial logistic regression analysis	Cancer (smoking, had blood relative with cancer, had a cancer history)	Cancer (perceived health status)	Cancer (cancer-related knowledge, discuss risk of cancer with doctor)	Cancer	80
57	Mckinney & Palmer 2014 [92]	USA	Descriptive, chi-square, t tests, multivariate logistic regression, multivariate linear regression	Colorectal cancer (gender)			Colorectal cancer	60
58	Zare Sakhvidi et al. 2014 [93]	Iran	t-test, linear regression			Occupational cancer (knowledge)	Occupational cancer	60
59	Chung & Lee 2013 [94]	Korea	Descriptive, chi-square test, ANOVA, analysis of covariance (ANCOVA)	Breast cancer (age, BMI)			Breast cancer	60
60	Mathur & Levy 2013 [95]	USA	Multivariate logistic regression	Lung cancer (smoker)	Lung cancer (distress)		Lung cancer	80
61	Metcalfe et al. 2013 [96]	Canada	Descriptive, analysis of variance, logistic regression analysis	Breast cancer (age, lifetime breast cancer risk estimation)			Breast cancer	60
62	Orom et al. 2013 [97]	USA	Chi-square tests, independent-sample t tests, linear regression, multinomial logistic regression	Breast cancer (ethnicity)			Breast cancer	80
63	Shiloh et al. 2013 [98]	USA	Pearson correlations, hierarchical regression analysis, principal component factor analyses	Osteoporosis (gender); Colorectal cancer (gender)	All studied NCDs (worry)		DM, osteoporosis, hypertension, coronary heart disease, hypercholesterolemia, skin cancer, lung cancer, colorectal cancer	60
64	Sudenga et al. 2013 [99]	Kenya	Descriptive, chi-square, t-test, stepwise regression	Cervical cancer (age, marital status)	Cervical cancer (feeling of adequately informed about cervical cancer by healthcare providers, intend to have cervical cancer screening in future)		Cervical cancer	60
65	Wang & Wu 2013 [100]	Taiwan	Descriptive, Pearson correlation			Cervical cancer (HPV vaccine knowledge)	Cervical cancer	60
66	Yang et al. 2013 [101]	China	Descriptive, chi-square, multiple logistic regression	Stroke (age, hypertension, hyperlipidemia, heart disease, previous stroke history)			Stroke	100
67	Buster et al. 2012 [102]	USA	Multiple logistic regression	Skin cancer (ethnicity, age, education)			Skin cancer	100
68	Chan & Leung 2012 [41]	Hong Kong	Descriptive, Mann-Whitney U test	Coronary heart disease (age, education)			Coronary heart disease	40
69	Darlow et al. 2012 [103]	USA	Multivariable logistic regression	DM (family history with DM); Heart disease (family history of heart disease)	DM (perceived one as overweight, believing overweight is a personal health problem; heart disease (perceived oneself as overweight)		DM, heart disease	60
70	Diaz et al. 2012 [104]	USA	Descriptive, t-test, chi-square, Fisher exact test, kappa statistics, stepwise logistic regression	DM (gender, age, BMI, reading ability, family history of DM); Heart attack (gender, hypertension, cholesterol level, family history of heart attack			DM, heart attack	40
71	Dickerson et al. 2012 [105]	USA	Bivariate ordered probit regression model, marginal probability analysis	DM (ethnicity, number of family members with DM)			DM	60
72	Haber et al. 2012 [106]	USA	Structural equation modeling framework, multinomial logistic regression	Breast cancer (family history of breast cancer, family history of cancer other than breast cancer)			Breast cancer	80
73	Hwang et al. 2012 [107]	Korea	Descriptive analysis, Pearson correlations, multiple linear regression	CVD (alcohol consumption, actual CVD risk, shift work)	CVD (perceived health status)	CVD (knowledge of CVD risk)	CVD	60
74	Kelly et al. 2012 [108]	USA	Descriptive, logistic regression analysis	Cervical cancer (history of sexually transmitted infection, smoking)	Cervical cancer (worry)	Cervical cancer (knowledge of cervical cancer)	Cervical cancer	80
75	White et al. 2012 [109]	USA	Pearson chi-square test, independent sample t tests, multivariate analysis of covariance (MANCOVA)	Head and neck cancer (smoking)			Head and neck cancer	60

Majority of the included studies were quantitative studies (84 cross-sectional studies and one cohort study), while one study was of mixed methods design. Most of the studies (n = 72) explored the associated factors of risk perception of NCDs, while 11 studies described the questionnaire for assessing risk perception of NCDs. Three studies describe both the questionnaire used to assess risk perception of NCDs, as well as the associated factors of risk perception of NCDs.

Table 2 shows the characteristics of the included studies related to questionnaire assessing risk perception of NCDs, including the measurement/s, study population, item development, analysis for validation and appraisal scoring. Table 3 shows the characteristics of the included studies related to associated factors of risk perception of NCDs, including the statistical analysis, associated factors, outcome (type of NCD) and quality appraisal scoring.

### Questionnaires measuring risk perception of NCDs

A total of six questionnaires were developed to measure risk perception of a group of NCDs, including CVDs, DM, cancer, chronic respiratory diseases, stroke, hypertension and cancer [31, 34–37, 40]. In other eight studies, the questionnaires were designed to measure risk perception of single NCD, including breast cancer [30, 32], cervical cancer [38], coronary heart disease [41], skin cancer [39], CVDs [29], CKD [33] and prostate cancer [28].

Of all the questionnaires, five were developed based on established theory or model such as the Health Belief Model and/or Transtheoretical Model [28, 31, 33, 36, 37]. A total of nine questionnaires were either adapted from previous questionnaires, developed based on literature review and/or expert opinion [29, 30, 32, 34, 35, 38–41]. All these questionnaires are available in a variety of languages such as English [34], Arabic [35], Yoruba [28], Malay [33], Chinese [41], Dutch [29] and Spanish [30].

All except four questionnaires [28, 30, 34, 37] explicitly assessed risk perception of NCDs. These four questionnaires are not intended only to measure risk perception of NCDs, but also focus on assessment of other components such as knowledge of NCDs and health behaviour [28, 34]. The total number of items in each of the included questionnaires ranges from six to 61, measured using Likert scales ranging from four to 10. The 5-point Likert scale is the most commonly used in the questionnaires.

In terms of validation, five questionnaires were only evaluated for either content validity and/or face validity [28, 34, 36, 38, 41]. A total of four studies used only exploratory factor analysis for validation of questionnaires [30, 37, 39, 40], while five questionnaires were well validated using both exploratory and confirmatory factory analysis [29, 31–33, 35].

### Associated factors of risk perception of NCDs

**Fig 2:**
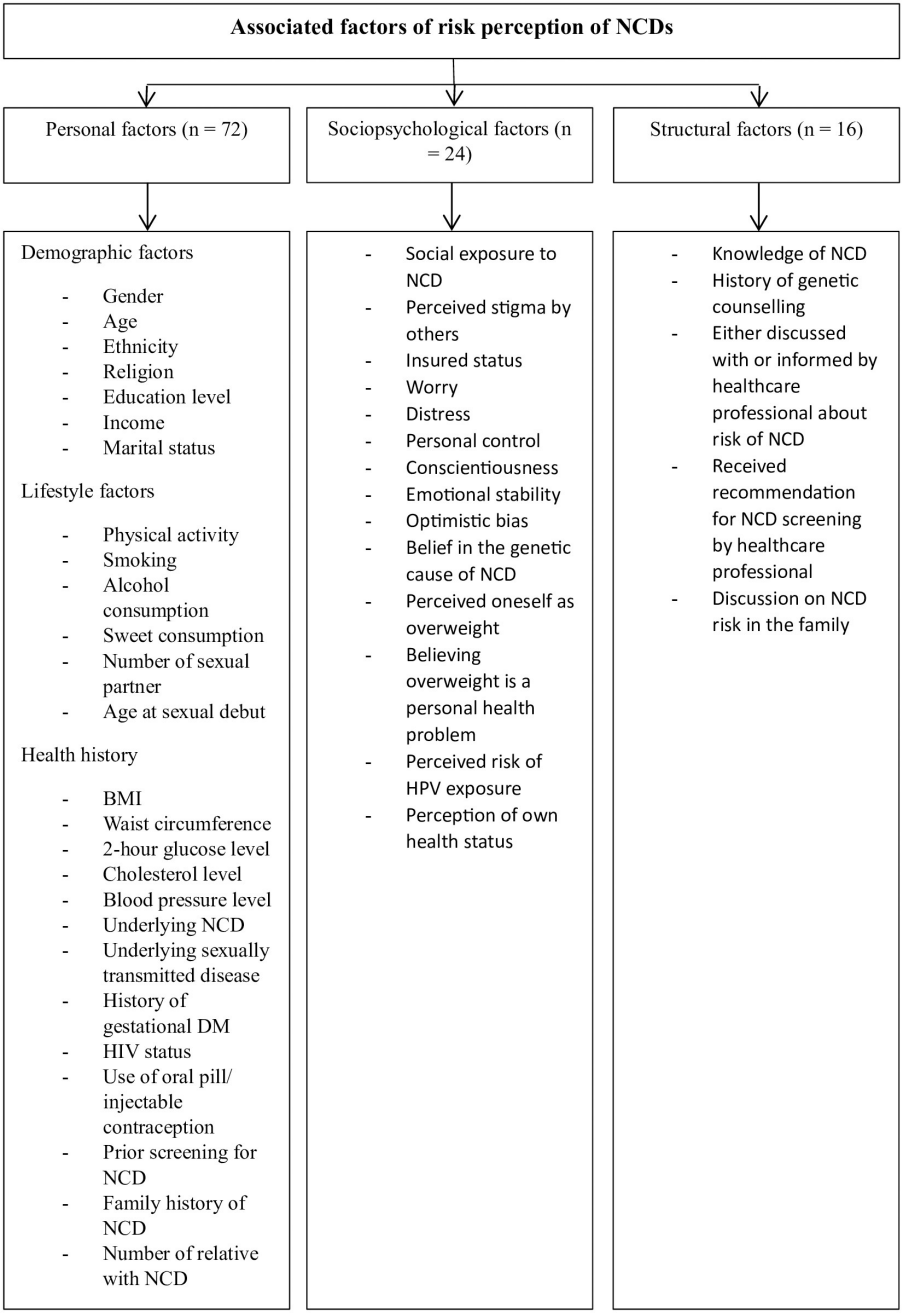
Associated factors of risk perception of NCDs.

Most of the studies (n = 72) reported associations between personal factors with risk perception of NCDs, including hypertension, DM, CVDs, CKD, cancers, stroke, osteoporosis and severe depression (Fig 2). A total of 24 studies reported associations between sociopsychological factors with risk perception of NCDs, while 16 studies reported associations between structural factors with risk perception of NCDs.

#### Personal factors

Personal factors including demographic, lifestyle and health history factors [14] were found to be associated with risk perception of NCDs. The identified demographic factors were gender, age, ethnicity, religion, education level, income and marital status. 16 studies examined associations between gender and risk perception of NCDs. A total of 30 and 12 studies reported associations between age and ethnicity with risk perception of NCDs.

Only one study each reported association between religion and marital status with NCD risk perception. A total of 11 studies reported associations between education level and NCD risk perception, while five studies found associations between income and risk perception of NCDs.

Several lifestyle factors were identified, including physical activity, smoking, alcohol consumption, sweet consumption, number of sexual partner and age at sexual debut. A total of five and ten studies reported associations between physical activity and smoking with NCD risk perception. Three and two studies reported associations between alcohol intake and sexual behaviour with NCD risk perception. Only one study reported association between sweets consumption and risk perception of DM.

The health history factors found to be associated with risk perception of NCDs include BMI, waist circumference, 2-hour glucose level, cholesterol level, blood pressure level, underlying NCD, underlying sexually transmitted disease, history of gestational DM, HIV status, use of oral pill/ injectable contraception, prior screening for NCD, family history of NCD and number of relative with NCD.

A total of 17 and two studies reported associations between BMI and waist circumference with NCD risk perception, respectively. Several studies (n = 14) reported associations between underlying comorbidities such as high blood pressure, high cholesterol, underlying NCDs, history of gestational DM, history of sexually transmitted disease and HIV positivity with risk perception of NCDs.

Only one study reported association between the use of hormonal contraception with risk perception of breast cancer. Three studies reported associations between prior screening of NCD with risk perception of NCD. A total of 31 studies reported associations between family history of NCD and/or number of relative with NCD with risk perception of NCDs.

#### Sociopsychological factors

Several sociopsychological factors were found to be associated with risk perception of NCDs, namely social exposure to NCD, perceived stigma by others, insured status, worry, distress, personal control, conscientiousness, emotional stability, optimistic bias, belief in the genetic cause of NCD, perceived oneself as overweight, believing overweight is a personal health problem, perceived risk of HPV exposure and perception of own health status.

Only one study reported association between social exposure to NCD and perception of stigma by other with risk perception of NCDs, while three studies found association between insured status and risk perception of NCDs. Emotional status such as worry and distress have been associated with risk perception of NCDs in five and one study, respectively.

Only one study found that greater personal control was associated with higher DM risk perception. Personality traits such as conscientiousness, emotional stability and optimistic were also found to be associated with risk perception of NCDs in three studies. Previous studies (n = 2) have occasionally examined health beliefs, while 11 studies reported associations between perception regarding own health and risk perception of NCDs.

#### Structural factors

In addition to personal and sociopsychological factors that may influence NCD risk perception, structural factors were also identified. Under structural factors, several knowledge-related factors were identified, such as knowledge of NCD (n = 7), history of genetic counselling (n = 1), either discussed with or informed by healthcare professional about risk of NCD (n = 4), received recommendation for NCD screening by healthcare professional (n = 1) and discussion on NCD risk in the family (n = 1).

### Critical appraisal of the included studies

The studies were appraised using the Mixed Methods Appraisal Tool (MMAT) [26]. The authors advised not to calculate overall score from the rating of each criterion using the latest version of MMAT (2018). However, due to problems faced by researchers in reporting the MMAT results, suggestion was provided for reporting of an overall score (5*****/100% quality criteria met; 4****/80% quality criteria met; 3***/60% quality criteria met; 2**/40% quality criteria met; 1*/20% quality criteria met). Of the included studies, 48 studies were given the score of 60%, while other 9, 25 and 4 studies were given the score of 100%, 80% and 40%, respectively. S2 File shows the scores of MMAT for each included study.

## Discussion

An individual’s risk perception of developing a disease is an important factor towards adopting healthy lifestyles and the uptake of preventive health interventions. To date, there has been no systematic review which has summarized the questionnaires assessing risk perception of NCDs and evaluated the evidence of factors influencing the risk perception of several major NCDs. Therefore, the purpose of this systematic review was to identify the best available evidence on questionnaire assessing risk perception of NCDs and factors associated with risk perception of NCDs. In this systematic review of 86 studies, we summarized the available questionnaire assessing risk perception of NCDs. We also identified several key personal, sociopsychological and structural factors associated with risk perception of NCDs.

### Questionnaires measuring risk perception of NCDs

This review has identified questionnaires that explicitly assessed risk perception of NCDs or included at least five items assessing risk perception of NCDs. These questionnaires that assessed risk perception of NCDs pose very different questions, as they were developed based on different model, theory or adapted from different studies. Nevertheless, most of these questionnaires comprehensively measure various dimensions of risk perception of NCDs given the multi-dimensional nature of the concept.

For example, questionnaire developed by Mya et al included five domains of risk perception, namely perceived susceptibility, perceived benefit, perceived barrier, self-efficacy and behavioural change intention [31], while another questionnaire included 6 domains, namely perceived susceptibility, perceived severity, perceived benefit, perceived barriers, perceived cues to action and perceived self-efficacy [36]. Similarly, in other study assessing multidimensional construct such as empowerment in the cancer population, a questionnaire including several different but related dimensions of empowerment was used [110]. The multiple dimensions of the included questionnaires can contribute to simultaneous data collection on multiple dimensions of NCD risk perception with reduced bias.

The strength of the questionnaires included in this review is their comprehensiveness within a relatively short questionnaire. For example, the 48-item questionnaire by Agarwal et al can be completed within 20 minutes which is acceptable without overloading the respondents [34]. Additionally, the questionnaire by Anuar et al that explicitly assessed risk perception of NCD contains the most items (61) among all included questionnaires [33]. Nevertheless, the completion time of this questionnaire is expected to be much shorter compared with other comprehensive survey that require up to two hours [111]. With the short duration of administration, the included questionnaires seem applicable in various settings including clinical and research settings.

Regarding the validity, only a few of the included questionnaires tested construct validity using confirmatory factor analysis. The lack of construct validity assessment is also commonly seen in questionnaires assessing other constructs such as empowerment in cancer patients and parenting style [112, 113]. Hence, more research is needed to understand whether the included questionnaires measure the NCD risk perception construct. In terms of mode of application, most of the included questionnaires used self-administered approach, which might improve their concurrent validity and reduce social desirability response bias as compared with interview approach [114]. Nevertheless, these questionnaires do not seem applicable in cross-country studies due to the validation in specific languages suitable for certain countries or region.

### Associated factors of risk perception of NCDs

Demographic factors including gender, age, ethnicity, religion, education level, income and marital status play a role in risk perception of NCDs. Even though many studies reported that women were more likely to perceived themselves to be at risk of NCDs [45, 80, 82], contradicting results have been reported [54, 88]. Sulaiman et al postulated that the lower risk perception of DM among women may be due to optimism bias, which is a cognitive bias that cause them to believe that they are less likely to experience a negative event [54].

Ample evidence exists regarding the association between age and risk perception of NCDs. The higher risk perception of NCDs among those of older age is explainable, as NCDs are often associated with older age due to the higher prevalence of NCDs in the older age group [1, 115]. Nevertheless, Kowall et al also suggested an explanation that older people perceive themselves to be at lower risk of NCDs due to their shorter life expectancy [67].

The association between ethnicity and risk perception of NCDs has been shown in the literature. The difference of risk perception between different ethnicities may be attributable to cultural salient factors such as wishful thinking, fatalistic beliefs and medical mistrust [61]. This finding highlights the need for further exploration of how cultural factors influence risk perception of NCDs. The higher risk perception of NCDs among people with higher educational attainment may be explained by the higher health literacy among people with higher educational attainment [116]. On the other hand, people with lower educational attainment may perceive themselves to be at risk of NCDs as they tend to be in the lower socioeconomic status group, which bears a disproportionate burden of NCDs [1].

The concerns of high socioeconomic status people about NCDs may be related to health literacy exposure, shaped by messages and media, as well as communication of health care professional [117]. However, those who had lower income may feel that they are more likely to be exposed to certain environmental hazards, which makes them feel vulnerable to cancer [85]. The higher risk perception of cervical cancer among married women may be due to the well-known fact that married women had higher risk of HPV infection hence at increased risk of cervical cancer [118].

Lifestyle factors associated with risk perception of NCDs included physical activity, smoking, alcohol consumption, sweet consumption and sexual behaviour. The knowledge regarding the benefit of physical activity in preventing NCDs might lead to lower risk perception among those who are physically active [15]. However, higher level of physical activity was not found to predict lower NCD risk perception in a cohort study, which might be due to their weakness in measurement of physical activity whereby different items were used at baseline and follow-up [46].

The higher risk perception of NCDs among smokers might be expected, as the adverse health effects of smoking is well known, particularly risk of cancers, CVD and respiratory diseases [119–121]. Similarly, the increased risk perception of NCDs accompanying alcohol consumption may reflect an appropriate awareness of the risk of alcohol consumption. Additionally, the positive association between sweets consumption and risk perception of DM may be explainable as the fact that consumption of sugar-containing food can lead to DM is well known [122, 123].

We also observed reported association between sexual behaviour and risk perception of NCDs. The higher risk perception of cervical cancer among those who had more sex partners is not surprising, as those who had more lifetime sexual partners and those practicing risky sexual lifestyle were more likely to be diagnosed with human papillomavirus infection which increases their risk of cervical cancer [64].

Health history factors associated with NCD risk perception included BMI, waist circumference, 2-hour glucose level, cholesterol level, blood pressure level, underlying NCD, underlying sexually transmitted disease, history of gestational DM, HIV status, use of oral pill/ injectable contraception, prior screening for NCD, family history of NCD and number of relative with NCD. The positive relationship between anthropometric values such as BMI and waist circumference with risk perception of NCD indicate that people seem to associate those readily visible factors with the risk of NCDs.

In addition to prior screening for NCD, underlying comorbidities such as high blood pressure, high cholesterol level, and pre-existing communicable and non-communicable diseases have also been associated with risk perception of NCDs. Those who had prior screening for NCD and underlying comorbidity may have more opportunity of receiving health counselling or advice from health care provider and therefore more likely to perceive risk for NCDs [83, 101].

The higher risk perception of breast cancer among those using hormonal contraception is possibly due to the established fact that hormonal contraception increases the risk of breast cancer [124]. There is also ample evidence to support the relationship between family history of NCDs and risk perception of NCDs. For certain NCDs such as DM and cancers, family history is among the important risk factors, which reflects an individual’s genetic predisposition for these NCDs [13, 125]. Therefore, it was not surprising that those with family history of NCDs perceived higher risk of developing those NCDs.

Sociopsychological factors including social exposure to NCD, perceived stigma by others, insured status, worry, distress, personal control, conscientiousness, emotional stability, optimistic bias, belief in the genetic cause of NCD, perceived oneself as overweight, believing overweight is a personal health problem, perceived risk of HPV exposure and perception of own health status may also play a role in NCD risk perception. The association between social exposure to NCD and personality traits (conscientiousness, emotional stability, optimistic) with risk perception of NCD is well supported by the Health Belief Model which suggested that prior contact of certain disease and personality can influence an individual’s risk perception of the disease [9].

The relationship between perception of stigmatized by other with risk perception of NCDs may be explained by the greater awareness of chronic disease among those who perceived stigma from others [84]. Studies also reported lower risk perception of NCDs among uninsured person, which may be a result of change in attitude as a form of self-protection. For example, those who are uninsured may feel that they cannot afford screening for cancer, causing them to push concerns about risk from their minds [71].

Emotional status such as worry and distress have been found to be associated with risk perception of NCDs. In view of these findings, future health interventions need to address NCD-related worry and distress to motivate health-related behaviours. Greater personal control was associated with higher risk perception of DM. However, this finding is contrary to the findings by Kim et al, which may be attributed to differences in the nature and composition of study sample [126]. Based on this literature, health belief may be important in risk perception of NCDs. This finding is in agreement with the Health Belief Model which proposed that sociopsychological factors can influence risk perception of an individual [9].

Literature also consistently reported association between perception regarding own health with risk perception of NCDs. The greater risk perception of DM among those who perceived themselves as overweight is possibly due to the higher health literacy, making them understand and aware that being overweight increases their risk for DM [103]. The lower risk perception of NCDs among those who perceived good health status warrants further studies to investigate effective methods to communicate risk of NCDs among this group of individuals especially those who are at high risk of NCDs.

Structural factors associated with NCD risk perception included several knowledge-related factors, namely knowledge of NCD, history of genetic counselling, either discussed with or informed by healthcare professional about risk of NCD, received recommendation for NCD screening by healthcare professional and discussion on NCD risk in the family. In general, it is not surprising that, those who were more knowledgeable about certain NCD had high risk perception of NCD. Furthermore the Health Belief Model also identified knowledge as a factor that can influence risk perception towards certain disease [9].

### Strengths and limitations

As with any research, this systematic review is not without limitations. Most of the included studies were of cross-sectional design, and therefore the quality of evidence was considered to be low and exhibit a high risk of bias. Another limitation relates to the measurement of risk perception of NCDs. Many studies assessed risk perception of NCDs using only a single item [46, 58, 82], which is insufficient to assess the multiple dimensions of the NCDs. Improving the measurement of risk perception of NCDs could help standardize the NCD risk perception literature.

The role of publication bias within this systematic review should be acknowledged as we did not include grey literature. We acknowledge the value of grey literature in systematic reviews. However, this systematic review did not include grey literature, in part due to the difficulty in searching and retrieving these documents, as well as time constraints. Another limitation is that we did not consult experts for source recommendations. Additionally, language bias must be considered as we only included English-language publications. Nevertheless, our search strategy has resulted in literature sourced from various countries where English is not the first language (China, Hong Kong, Italy, Spain). One of the strengths of this review is that we have included both questionnaires that explicitly assessed risk perception of NCDs, as well as questionnaires that included at least five items related to risk perception of NCDs. This process increased the chances of identifying items related to risk perception of NCDs for future research. Additionally, to the best of our knowledge, this is the first systematic review synthetizing research evidence on risk perception of several major NCDs, highlighting the available questionnaire assessing NCD risk perception and the correlates of perceived risk of acquiring NCDs.

## Conclusion

This systematic review may serve as a guideline for researchers who intend to measure risk perception of NCDs, as the results provide an overview of the components included in those questionnaires, which can provide guidance in the choice of questionnaire. Nevertheless, further evidence is needed to support the validity of these questionnaires. In view of this limitation, researchers should pay attention in assessing validity in future questionnaire development work.

This systematic review also highlighted several key factors related to the risk perception of NCDs. All these key factors deserve further exploration in longitudinal and experimental studies. Based on the synthesized evidence, health interventions addressing risk perception of NCDs should not only focus on facts about personal factors (demographic, lifestyle and health history factors), but also on the sociopsychological and structural factors that may influence the risk perception of NCDs particularly among those at risk of NCDs.

## Supporting information

S1 Appendix PRISMA 2020 checklist. (DOCX)

S1 File Search strategy. (DOCX)

S2 File Critical appraisal of selected studies using MMAT. (DOCX)

S1 Table Data. (XLSX)
