# Supplementary material for: Risk perception of non-communicable diseases: A systematic review on its assessment and associated factors
Source: PLoS One. 2023 Jun 1;18(6):e0286518. doi: 10.1371/journal.pone.0286518 (PMC10234567; doi:10.1371/journal.pone.0286518)
Supplement: S1 File — (DOCX) [file pone.0286518.s002.docx]

**Search strategy**

| Objective | To identify available questionnaire for assessing risk perception of NCDs and to characterize the existing literatures on factors associated with risk perception of NCDs |
| --- | --- |
| Literature type | Published articles |
| Study settings | Global |
| Inclusion criteria | (1) Publication from 2012-2021  (2) Publication in English language  (3) Original article  (4) Described questionnaire that explicitly assessed risk perception of non-communicable diseases or questionnaire that contained at least five items measuring risk perception of non-communicable diseases  (5) Described associated factors of risk perception of non-communicable diseases |
| Exclusion criteria | Non-original articles including conference proceedings, commentary, reports, review articles and systematic reviews |
| Databases | Scopus, Web of Science, PubMed |
| Languages | English only |
| Search period | Searches was performed in December 2021 |

**Database search terms**

| **Database** | **Keywords** | **Filters** |
| --- | --- | --- |
| Scopus | 1) TITLE-ABS-KEY(("questionnaire" OR "instrument" OR "tool") AND ("risk perception" OR "perceived risk") AND ("non-communicable disease*" OR "noncommunicable disease*" OR "NCD" OR "chronic respiratory disease*" OR "chronic obstructive pulmonary disease*" OR "asthma" OR "hypertension" OR "diabetes" OR "diabetes mellitus" OR "cancer" OR "neoplasm", "cardiovascular disease" OR "heart attack" OR "stroke"))  2) TITLE-ABS-KEY((“associated factor*” OR “risk factor*” OR “association*” OR “determinant*” OR “predictor*”) AND (“risk perception” OR “perceived risk”) AND ("non-communicable disease*" OR "noncommunicable disease*" OR "NCD" OR "chronic respiratory disease*" OR "chronic obstructive pulmonary disease*" OR "asthma" OR "hypertension" OR "diabetes" OR "diabetes mellitus" OR "cancer" OR "neoplasm", "cardiovascular disease" OR "heart attack" OR "stroke")) | (1) Year: 2012-2021  (2) Language: English  (3) Document type: article |
| Web of Science | 1) TS=(("questionnaire" OR "instrument" OR "tool") AND ("risk perception" OR "perceived risk") AND ("non-communicable disease*" OR "noncommunicable disease*" OR "NCD" OR "chronic respiratory disease*" OR "chronic obstructive pulmonary disease*" OR "asthma" OR "hypertension" OR "diabetes" OR "diabetes mellitus" OR "cancer" OR "neoplasm", "cardiovascular disease" OR "heart attack" OR "stroke"))  2) TS=((“associated factor*” OR “risk factor*” OR “association*” OR “determinant*” OR “predictor*”) AND (“risk perception” OR “perceived risk”) AND ("non-communicable disease*" OR "noncommunicable disease*" OR "NCD" OR "chronic respiratory disease*" OR "chronic obstructive pulmonary disease*" OR "asthma" OR "hypertension" OR "diabetes" OR "diabetes mellitus" OR "cancer" OR "neoplasm", "cardiovascular disease" OR "heart attack" OR "stroke")) | (1) Year: 2012-2021  (2) Language: English  (3) Document type: article |
| PubMed | 1) ((questionnaire[Title/Abstract] OR instrument[Title/Abstract] OR tool[Title/Abstract]) AND (risk perception[Title/Abstract] OR perceived risk[Title/Abstract])) AND (non-communicable disease*[Title/Abstract] OR noncommunicable disease*[Title/Abstract] OR NCD[Title/Abstract] OR chronic respiratory disease*[Title/Abstract] OR chronic obstructive pulmonary disease*[Title/Abstract] OR asthma[Title/Abstract] OR hypertension[Title/Abstract] OR diabetes[Title/Abstract] OR diabetes mellitus[Title/Abstract] OR cancer[Title/Abstract] OR neoplasm[Title/Abstract] OR cardiovascular disease[Title/Abstract] OR heart attack[Title/Abstract] OR stroke[Title/Abstract])  2) ((associated factor*[Title/Abstract] OR risk factor*[Title/Abstract] OR association*[Title/Abstract] OR determinant*[Title/Abstract] OR predictor*[Title/Abstract]) AND (risk perception[Title/Abstract] OR perceived risk[Title/Abstract])) AND (non-communicable disease*[Title/Abstract] OR noncommunicable disease*[Title/Abstract] OR NCD[Title/Abstract] OR chronic respiratory disease*[Title/Abstract] OR chronic obstructive pulmonary disease*[Title/Abstract] OR asthma[Title/Abstract] OR hypertension[Title/Abstract] OR diabetes[Title/Abstract] OR diabetes mellitus[Title/Abstract] OR cancer[Title/Abstract] OR neoplasm[Title/Abstract] OR cardiovascular disease[Title/Abstract] OR heart attack[Title/Abstract] OR stroke[Title/Abstract]) | (1) Year: 2012-2021  (2) Language: English |
