# Supplementary material for: Risk perception of non-communicable diseases: A systematic review on its assessment and associated factors
Source: PLoS One. 2023 Jun 1;18(6):e0286518. doi: 10.1371/journal.pone.0286518 (PMC10234567; doi:10.1371/journal.pone.0286518)
Supplement: S2 File — (DOCX) [file pone.0286518.s003.docx]

**Critical appraisal of selected studies using MMAT**

| **Category of study designs** | **Methodological quality criteria** |
| --- | --- |
| 1. Qualitative | 1.1 Is the qualitative approach appropriate to answer the research question? |
|  | 1.2 Are the qualitative data collection methods adequate to address the research question? |
|  | 1.3 Are the findings adequately derived from the data? |
|  | 1.4 Is the interpretation of results sufficiently substantiated by data? |
|  | 1.5 Is there coherence between qualitative data sources, collection, analysis and interpretation? |
| 2. Quantitative randomized controlled trials | 2.1 Is randomization appropriately performed? |
|  | 2.2 Are the groups comparable at baseline? |
|  | 2.3 Are there complete outcome data? |
|  | 2.4 Are outcome assessors blinded to the intervention provided? |
|  | 2.5 Did the participants adhere to the assigned intervention? |
| 3. Quantitative non-randomized | 3.1 Are the participants representative of the target population? |
|  | 3.2 Are measurements appropriate regarding both the outcome and intervention (or exposure)? |
|  | 3.3 Are there complete outcome data? |
|  | 3.4 Are the confounders accounted for in the design and analysis? |
|  | 3.5 During the study period, is the intervention administered (or exposure occurred) as intended? |
| 4. Quantitative descriptive | 4.1 Is the sampling strategy relevant to address the research question? |
|  | 4.2 Is the sample representative of the target population? |
|  | 4.3 Are the measurements appropriate? |
|  | 4.4 Is the risk of nonresponse bias low? |
|  | 4.5 Is the statistical analysis appropriate to answer the research question? |
| 5. Mixed methods | 5.1 Is there an adequate rationale for using a mixed methods design to address the research question? |
|  | 5.2 Are the different components of the study effectively integrated to answer the research question? |
|  | 5.3 Are the outputs of the integration of qualitative and quantitative components adequately interpreted? |
|  | 5.4 Are divergences and inconsistencies between quantitative and qualitative results adequately addressed? |
|  | 5.5 Do the different components of the study adhere to the quality criteria of each tradition of the methods involved? |

| **No** | **Author, Year** | **The Mixed Methods Appraisal Tool Criteria** | | | | | | | | | | | | | | | | | | | | | | | | | **Quality (%)** | |  |
| --- | --- | --- | --- | --- | --- | --- | --- | --- | --- | --- | --- | --- | --- | --- | --- | --- | --- | --- | --- | --- | --- | --- | --- | --- | --- | --- | --- | --- | --- |
|  |  | **1.1** | **1.2** | **1.3** | **1.4** | **1.5** | **2.1** | **2.2** | **2.3** | **2.4** | **2.5** | **3.1** | **3.2** | **3.3** | **3.4** | **3.5** | **4.1** | **4.2** | **4.3** | **4.4** | **4.5** | **5.1** | **5.2** | **5.3** | **5.4** | **5.5** | |  | |
| 1 | Adedeji et al. 2021 |  |  |  |  |  |  |  |  |  |  |  |  |  |  |  | yes | yes | yes | yes | yes |  |  |  |  |  | | 100 | |
| 2 | Fein et al. 2021 |  |  |  |  |  |  |  |  |  |  |  |  |  |  |  | yes | no | yes | no | yes |  |  |  |  |  | | 60 | |
| 3 | Grauman et al. 2021 |  |  |  |  |  |  |  |  |  |  |  |  |  |  |  | yes | yes | yes | no | yes |  |  |  |  |  | | 80 | |
| 4 | Hassen et al. 2021 |  |  |  |  |  |  |  |  |  |  |  |  |  |  |  | yes | yes | yes | no | yes |  |  |  |  |  | | 80 | |
| 5 | Martínez-Urquijo et al. 2021 |  |  |  |  |  |  |  |  |  |  |  |  |  |  |  | no | yes | yes | no | no |  |  |  |  |  | | 40 | |
| 6 | Mya et al. 2021 | yes | yes | yes | yes | yes |  |  |  |  |  |  |  |  |  |  | yes | yes | yes | yes | yes | yes | yes | yes | yes | yes | | 100 | |
| 7 | Osei et al. 2021 |  |  |  |  |  |  |  |  |  |  |  |  |  |  |  | yes | yes | yes | no | yes |  |  |  |  |  | | 80 | |
| 8 | Taylan et al. 2021 |  |  |  |  |  |  |  |  |  |  |  |  |  |  |  | no | yes | yes | no | yes |  |  |  |  |  | | 60 | |
| 9 | Turner et al. 2021 |  |  |  |  |  |  |  |  |  |  |  |  |  |  |  | yes | no | yes | no | yes |  |  |  |  |  | | 60 | |
| 10 | Vornanen et al. 2021 |  |  |  |  |  |  |  |  |  |  |  |  |  |  |  | yes | yes | yes | yes | yes |  |  |  |  |  | | 100 | |
| 11 | Zarghami et al. 2021 |  |  |  |  |  |  |  |  |  |  |  |  |  |  |  | yes | yes | yes | no | yes |  |  |  |  |  | | 80 | |
| 12 | Abshire et al. 2020 |  |  |  |  |  |  |  |  |  |  |  |  |  |  |  | no | yes | yes | no | yes |  |  |  |  |  | | 60 | |
| 13 | Antwi et al. 2020 |  |  |  |  |  |  |  |  |  |  |  |  |  |  |  | yes | no | yes | no | yes |  |  |  |  |  | | 60 | |
| 14 | Anuar et al. 2020 |  |  |  |  |  |  |  |  |  |  |  |  |  |  |  | no | yes | yes | yes | yes |  |  |  |  |  | | 80 | |
| 15 | Khan et al. 2020 |  |  |  |  |  |  |  |  |  |  |  |  |  |  |  | yes | no | yes | yes | yes |  |  |  |  |  | | 80 | |
| 16 | Khlaifat et al. 2020 |  |  |  |  |  |  |  |  |  |  |  |  |  |  |  | yes | yes | yes | no | yes |  |  |  |  |  | | 80 | |
| 17 | Palmero et al. 2020 |  |  |  |  |  |  |  |  |  |  |  |  |  |  |  | yes | no | yes | no | yes |  |  |  |  |  | | 60 | |
| 18 | Russell et al. 2020 |  |  |  |  |  |  |  |  |  |  |  |  |  |  |  | yes | no | yes | no | yes |  |  |  |  |  | | 60 | |
| 19 | Stol et al. 2020 |  |  |  |  |  |  |  |  |  |  |  |  |  |  |  | yes | no | yes | no | yes |  |  |  |  |  | | 60 | |
| 20 | Sulaiman et al. 2020 |  |  |  |  |  |  |  |  |  |  |  |  |  |  |  | yes | yes | yes | no | yes |  |  |  |  |  | | 80 | |
| 21 | Agarwal et al. 2019 |  |  |  |  |  |  |  |  |  |  |  |  |  |  |  | no | yes | yes | no | yes |  |  |  |  |  | | 60 | |
| 22 | Gibson et al. 2019 |  |  |  |  |  |  |  |  |  |  |  |  |  |  |  | yes | no | yes | yes | yes |  |  |  |  |  | | 80 | |
| 23 | Guo et al. 2019 |  |  |  |  |  |  |  |  |  |  |  |  |  |  |  | yes | no | yes | no | yes |  |  |  |  |  | | 60 | |
| 24 | Hall et al. 2019 |  |  |  |  |  |  |  |  |  |  |  |  |  |  |  | yes | yes | yes | yes | yes |  |  |  |  |  | | 100 | |
| 25 | Heidemann et al. 2019 |  |  |  |  |  |  |  |  |  |  |  |  |  |  |  | yes | no | yes | no | yes |  |  |  |  |  | | 60 | |
| 26 | Hsueh et al. 2019 |  |  |  |  |  |  |  |  |  |  |  |  |  |  |  | yes | yes | yes | yes | yes |  |  |  |  |  | | 100 | |
| 27 | Pelullo et al. 2019 |  |  |  |  |  |  |  |  |  |  |  |  |  |  |  | yes | yes | yes | yes | yes |  |  |  |  |  | | 100 | |
| 28 | Perez et al. 2019 |  |  |  |  |  |  |  |  |  |  |  |  |  |  |  | yes | no | yes | no | yes |  |  |  |  |  | | 60 | |
| 29 | Turbitt et al. 2019 |  |  |  |  |  |  |  |  |  |  |  |  |  |  |  | yes | no | yes | no | yes |  |  |  |  |  | | 60 | |
| 30 | Alaa & Shah 2018 |  |  |  |  |  |  |  |  |  |  |  |  |  |  |  | no | yes | yes | no | yes |  |  |  |  |  | | 60 | |
| 31 | Chalian et al. 2018 |  |  |  |  |  |  |  |  |  |  |  |  |  |  |  | yes | yes | yes | no | yes |  |  |  |  |  | | 80 | |
| 32 | Pasi et al. 2018 |  |  |  |  |  |  |  |  |  |  |  |  |  |  |  | yes | no | yes | no | yes |  |  |  |  |  | | 60 | |
| 33 | Skøt et al. 2018 |  |  |  |  |  |  |  |  |  |  |  |  |  |  |  | yes | no | yes | no | yes |  |  |  |  |  | | 60 | |
| 34 | Wilson et al. 2018 |  |  |  |  |  |  |  |  |  |  |  |  |  |  |  | yes | no | yes | no | yes |  |  |  |  |  | | 60 | |
| 35 | Butler et al. 2017 |  |  |  |  |  |  |  |  |  |  |  |  |  |  |  | yes | no | yes | no | yes |  |  |  |  |  | | 60 | |
| 36 | Chopra & Chopra 2017 |  |  |  |  |  |  |  |  |  |  |  |  |  |  |  | yes | no | yes | no | yes |  |  |  |  |  | | 60 | |
| 37 | Desgraz et al. 2017 |  |  |  |  |  |  |  |  |  |  |  |  |  |  |  | yes | no | yes | yes | yes |  |  |  |  |  | | 80 | |
| 38 | Kaba et al. 2017 |  |  |  |  |  |  |  |  |  |  |  |  |  |  |  | yes | no | yes | no | yes |  |  |  |  |  | | 60 | |
| 39 | Kowall et al. 2017 |  |  |  |  |  |  |  |  |  |  |  |  |  |  |  | yes | no | yes | no | yes |  |  |  |  |  | | 60 | |
| 40 | Osazuwa-Peters et al. 2017 |  |  |  |  |  |  |  |  |  |  |  |  |  |  |  | yes | no | yes | no | yes |  |  |  |  |  | | 60 | |
| 41 | Silverman et al. 2017 |  |  |  |  |  |  |  |  |  |  |  |  |  |  |  | yes | yes | yes | no | yes |  |  |  |  |  | | 80 | |
| 42 | Taber et al. 2017 |  |  |  |  |  |  |  |  |  |  |  |  |  |  |  | yes | no | yes | yes | yes |  |  |  |  |  | | 80 | |
| 43 | Vandyke & Shell 2017 |  |  |  |  |  |  |  |  |  |  |  |  |  |  |  | yes | no | yes | no | yes |  |  |  |  |  | | 60 | |
| 44 | Woringer et al. 2017 |  |  |  |  |  |  |  |  |  |  |  |  |  |  |  | yes | yes | yes | yes | no |  |  |  |  |  | | 80 | |
| 45 | Yüksel et al. 2017 |  |  |  |  |  |  |  |  |  |  |  |  |  |  |  | yes | yes | yes | no | yes |  |  |  |  |  | | 80 | |
| 46 | Basilio et al. 2016 |  |  |  |  |  |  |  |  |  |  |  |  |  |  |  | yes | no | yes | no | yes |  |  |  |  |  | | 60 | |
| 47 | Boo et al. 2016 |  |  |  |  |  |  |  |  |  |  |  |  |  |  |  | yes | no | yes | no | yes |  |  |  |  |  | | 60 | |
| 48 | Chen et al. 2016 |  |  |  |  |  |  |  |  |  |  |  |  |  |  |  | yes | yes | yes | no | yes |  |  |  |  |  | | 80 | |
| 49 | Joiner et al. 2016 |  |  |  |  |  |  |  |  |  |  |  |  |  |  |  | yes | no | yes | no | yes |  |  |  |  |  | | 60 | |
| 50 | Lin et al. 2016 |  |  |  |  |  |  |  |  |  |  |  |  |  |  |  | no | no | yes | yes | yes |  |  |  |  |  | | 60 | |
| 51 | Osazuwa-Peters & Tutlam 2016 |  |  |  |  |  |  |  |  |  |  |  |  |  |  |  | yes | no | yes | no | yes |  |  |  |  |  | | 60 | |
| 52 | Shah et al. 2016 |  |  |  |  |  |  |  |  |  |  |  |  |  |  |  | yes | no | yes | no | yes |  |  |  |  |  | | 60 | |
| 53 | Vornanen et al. 2016 |  |  |  |  |  |  |  |  |  |  |  |  |  |  |  | yes | yes | yes | yes | yes |  |  |  |  |  | | 100 | |
| 54 | Amuta et al. 2015 |  |  |  |  |  |  |  |  |  |  |  |  |  |  |  | yes | no | yes | no | yes |  |  |  |  |  | | 60 | |
| 55 | Clark & Lavielle 2015 |  |  |  |  |  |  |  |  |  |  |  |  |  |  |  | yes | yes | yes | no | yes |  |  |  |  |  | | 80 | |
| 56 | Fukuoka et al. 2015 |  |  |  |  |  |  |  |  |  |  |  |  |  |  |  | yes | no | yes | yes | yes |  |  |  |  |  | | 80 | |
| 57 | Guess et al. 2015 |  |  |  |  |  |  |  |  |  |  |  |  |  |  |  | yes | no | yes | no | yes |  |  |  |  |  | | 60 | |
| 58 | Hamilton & Lobel 2015 |  |  |  |  |  |  |  |  |  |  |  |  |  |  |  | no | no | yes | no | yes |  |  |  |  |  | | 40 | |
| 59 | Kye et al. 2015 |  |  |  |  |  |  |  |  |  |  |  |  |  |  |  | yes | no | yes | no | yes |  |  |  |  |  | | 60 | |
| 60 | Peipins et al. 2015 |  |  |  |  |  |  |  |  |  |  |  |  |  |  |  | yes | no | yes | no | yes |  |  |  |  |  | | 60 | |
| 61 | Piccinino et al. 2015 |  |  |  |  |  |  |  |  |  |  |  |  |  |  |  | yes | yes | yes | no | yes |  |  |  |  |  | | 80 | |
| 62 | Rice et al. 2015 |  |  |  |  |  |  |  |  |  |  |  |  |  |  |  | yes | yes | yes | no | yes |  |  |  |  |  | | 80 | |
| 63 | Temu et al. 2015 |  |  |  |  |  |  |  |  |  |  |  |  |  |  |  | yes | no | yes | no | yes |  |  |  |  |  | | 60 | |
| 64 | Godino et al. 2014 |  |  |  |  |  |  |  |  |  |  |  |  |  |  |  | yes | no | yes | no | yes |  |  |  |  |  | | 60 | |
| 65 | Lucas-Wright et al. 2014 |  |  |  |  |  |  |  |  |  |  |  |  |  |  |  | yes | no | yes | yes | yes |  |  |  |  |  | | 80 | |
| 66 | Mckinney & Palmer 2014 |  |  |  |  |  |  |  |  |  |  |  |  |  |  |  | yes | no | yes | no | yes |  |  |  |  |  | | 60 | |
| 67 | Morales-Sánchez et al. 2014 |  |  |  |  |  |  |  |  |  |  |  |  |  |  |  | no | yes | yes | no | yes |  |  |  |  |  | | 60 | |
| 68 | Zare Sakhvidi et al. 2014 |  |  |  |  |  |  |  |  |  |  |  |  |  |  |  | yes | no | yes | no | yes |  |  |  |  |  | | 60 | |
| 69 | Chung & Lee 2013 |  |  |  |  |  |  |  |  |  |  |  |  |  |  |  | no | yes | yes | no | yes |  |  |  |  |  | | 60 | |
| 70 | Hafizah et al. 2013 |  |  |  |  |  |  |  |  |  |  |  |  |  |  |  | no | yes | yes | no | yes |  |  |  |  |  | | 60 | |
| 71 | Mathur & Levy 2013 |  |  |  |  |  |  |  |  |  |  |  |  |  |  |  | yes | yes | yes | no | yes |  |  |  |  |  | | 80 | |
| 72 | Metcalfe et al. 2013 |  |  |  |  |  |  |  |  |  |  |  |  |  |  |  | yes | no | yes | no | yes |  |  |  |  |  | | 60 | |
| 73 | Orom et al. 2013 |  |  |  |  |  |  |  |  |  |  |  |  |  |  |  | yes | yes | yes | no | yes |  |  |  |  |  | | 80 | |
| 74 | Shiloh et al. 2013 |  |  |  |  |  |  |  |  |  |  |  |  |  |  |  | yes | no | yes | no | yes |  |  |  |  |  | | 60 | |
| 75 | Sudenga et al. 2013 |  |  |  |  |  |  |  |  |  |  |  |  |  |  |  | yes | no | yes | no | yes |  |  |  |  |  | | 60 | |
| 76 | Wang & Wu 2013 |  |  |  |  |  |  |  |  |  |  |  |  |  |  |  | yes | no | yes | no | yes |  |  |  |  |  | | 60 | |
| 77 | Yang et al. 2013 |  |  |  |  |  |  |  |  |  |  |  |  |  |  |  | yes | yes | yes | yes | yes |  |  |  |  |  | | 100 | |
| 78 | Buster et al. 2012 |  |  |  |  |  |  |  |  |  |  |  |  |  |  |  | yes | yes | yes | yes | yes |  |  |  |  |  | | 100 | |
| 79 | Chan & Leung 2012 |  |  |  |  |  |  |  |  |  |  |  |  |  |  |  | no | no | yes | no | yes |  |  |  |  |  | | 40 | |
| 80 | Darlow et al. 2012 |  |  |  |  |  |  |  |  |  |  |  |  |  |  |  | yes | no | yes | no | yes |  |  |  |  |  | | 60 | |
| 81 | Diaz et al. 2012 |  |  |  |  |  |  |  |  |  |  |  |  |  |  |  | no | no | yes | no | yes |  |  |  |  |  | | 40 | |
| 82 | Dickerson et al. 2012 |  |  |  |  |  |  |  |  |  |  |  |  |  |  |  | yes | no | yes | no | yes |  |  |  |  |  | | 60 | |
| 83 | Haber et al. 2012 |  |  |  |  |  |  |  |  |  |  |  |  |  |  |  | yes | yes | yes | no | yes |  |  |  |  |  | | 80 | |
| 84 | Hwang et al. 2012 |  |  |  |  |  |  |  |  |  |  |  |  |  |  |  | yes | no | yes | no | yes |  |  |  |  |  | | 60 | |
| 85 | Kelly et al. 2012 |  |  |  |  |  |  |  |  |  |  |  |  |  |  |  | yes | yes | yes | no | yes |  |  |  |  |  | | 80 | |
| 86 | White et al. 2012 |  |  |  |  |  |  |  |  |  |  |  |  |  |  |  | yes | no | yes | no | yes |  |  |  |  |  | | 60 | |
